# Research Centers Collaborative Network Workshop on Digital Health Approaches to Research in Aging

**DOI:** 10.1093/geroni/igae012

**Published:** 2024-02-08

**Authors:** Jason Fanning, Tina E Brinkley, Laura M Campbell, Cristina Colon-Semenza, Sara J Czaja, Raeanne C Moore, Nicholas M Pajewski, Stephen Kritchevsky

**Affiliations:** Department of Health and Exercise Science, Wake Forest University, Winston-Salem, North Carolina, USA; Department of Internal Medicine, Gerontology and Geriatric Medicine, Wake Forest University School of Medicine, Winston-Salem, North Carolina, USA; Department of Psychiatry, University of California San Diego School of Medicine, San Diego, California, USA; Department of Kinesiology, University of Connecticut, Storrs, Connecticut, USA; Division of Geriatrics and Palliative Medicine, Weill Cornell Medicine, New York City, New York, USA; Department of Psychiatry, University of California San Diego School of Medicine, San Diego, California, USA; Department of Biostatistics and Data Science, Wake Forest University School of Medicine, Winston-Salem, North Carolina, USA; Department of Internal Medicine, Gerontology and Geriatric Medicine, Wake Forest University School of Medicine, Winston-Salem, North Carolina, USA

**Keywords:** Behavioral intervention, Digital health, Ecological momentary assessment, eHealth, mHealth

## Abstract

Digital health technologies are ubiquitous in the healthcare landscape. Older adults represent an important user group who may benefit from improved monitoring of physical and cognitive health and in-home access to care, but there remain many barriers to widespread use of digital health technologies in gerontology and geriatric medicine. The National Institute on Aging Research Centers Collaborative Network convened a workshop wherein geriatricians and gerontological researchers with expertise related to mHealth and digital health applications shared opportunities and challenges in the application of digital health technologies in aging. Discussion broadly centered on 2 themes: promises and challenges in (i) the use of ecological momentary assessment methodologies in gerontology and geriatric medicine, and (ii) the development of health promotion programs delivered via digital health technologies. Herein, we summarize this discussion and outline several promising areas for future research.

**Translational Significance:** Digital health technologies present opportunities and challenges to the care of older adults. These tools can extend care into the home and guide decision making based on data collected in the context in which people live. Conversely, how best to design these tools to meet the needs and preferences of older adults and those who care for them, and how to leverage data produced via digital health technologies to guide care remain key challenges. This article summarizes discussion arising from a workshop on digital health approaches to research in aging and provides recommendations for clinical care and future research.

Digital health is ubiquitous within the healthcare landscape, a phenomenon accelerated by the coronavirus disease 2019 (COVID-19) pandemic (1). Digital health can be understood as the field of knowledge and practice associated with the development and use of digital technologies to improve health (2). This definition encompasses the terms *eHealth* and *mHealth*, where eHealth describes the provision of health services via information and communication technology, and often the term is used to describe internet- or computer-delivered services. mHealth refers specifically to the use of mobile technologies, and is commonly used to describe health-oriented smartphone or tablet applications (3). Digital health further describes other uses of digital technology to support health, using technologies as diverse as machine learning and big data analytics to the “internet of things.” These tools offer daily or even within-day insight into important psychological, social, and physical determinants of health, and they extend the reach of traditionally center-based behavior change programming into the home.

Older adults are a particularly important digital health user group. Digital health technologies promise the ability to reach those with limitations in their ability to access in-person care (eg, due to lack of access to transport or geographic or social isolation). Moreover, these tools can provide continuous, autonomous monitoring of threats to health and safety such as fall detection via accelerometry, restricted life space via motion sensors, or indications of cognitive decline (eg, leaving a stove on), thereby assisting older adults in aging in place. While the field of digital health is rapidly advancing, in many ways, the research and clinical application of these technologies is in its nascency, with many barriers to widespread use remaining. In November 2022, the National Institute on Aging Research Centers Collaborative Network (RCCN) convened a workshop (4) on the application of digital health technologies in aging research. Workshop attendees and presenters included geriatricians and gerontological researchers with expertise in clinical, behavioral, measurement, informatic, and statistical considerations related to mHealth and digital health applications. Two key areas of interest arose naturally both in presentation and in discussion, including promises and challenges in (i) the use of ecological momentary assessment (EMA) methodologies (5) in gerontology and geriatric medicine, and (ii) the development of health promotion programs via eHealth and mHealth technologies. What follows is a summary of key points of discussion related to these 2 themes.

## Considerations in the Measurement of Patient-Reported Outcomes in Daily Life and in Real Time

Widespread access to internet and mobile technologies has provided researchers and clinicians with unprecedented opportunities to measure health-related phenomena at increasingly fine resolution and over longer timescales. This is especially true of the ability to measure psychosocial phenomena—which are often highly dynamic and capture subjective states—in the real world and in real time. A major theme of the workshop centered on the well-established advantages of EMA methodologies in the context of aging research, and the emerging research gaps that widespread use of these strategies presents.

### What Is EMA?

Contemporary conceptualizations of successful aging consider a biopsychosocial approach that recognizes that poor health is driven by interacting physical, social, and psychological determinants (6). In traditional clinical and research contexts, valid and reliable objective (eg, cognitive performance, functional performance) and self-reported (eg, affect, stress) measures are either not ascertained unless prompted for a clinical diagnosis, or are collected at infrequent intervals, often with the aim of characterizing a given phenomenon over a span of time (7). Such an approach is subject to important limitations, impeding the understanding of the causes and consequences of health and health behaviors. For instance, researchers and clinicians are often interested in the dynamics of a psychosocial health determinant (eg, a momentary peak in pain or stress) relative to one’s “average” experience of the phenomenon. EMA strategies aim to capture one’s experiences in the real world and in real time (5). EMA protocols often entail repeated daily assessments of patient-reported data, allowing researchers to investigate dynamic within-person relationships between psychosocial states and health outcomes of interest. For instance, Phillips and colleagues (8) investigated the relationships between EMA-reported breast cancer treatment-related symptoms and objective physical activity behaviors in a sample of 67 women undergoing chemotherapy. The authors demonstrated that daily fluctuations around one’s average pain, fatigue, and daily functioning ratings were associated with physical activity behaviors on that day as well as on the following day.

Infrequent self-report summary assessments are also subject to biases that affect the extent to which one’s rating reflects their lived experience. For instance, individual personality traits powerfully affect the accuracy of one’s recall, with traits like neuroticism or extraversion contributing to exaggerated ratings of distress and positive affect, respectively, relative to diary reports (9). The duration over which a person is asked to reflect also affects the memories an individual calls upon to answer a given item. Winkielman et al. (10) demonstrated that when asked about how often one felt angry over the previous year, participants called on rare but intense periods of anger to produce their response. By contrast, when queried about anger in the previous week, participants referenced more common and less intense experiences in forming their response. Interestingly, the dynamics of a health determinant, such as within-day or day-to-day variation in a construct, also affect the accuracy of ratings. Stone and colleagues demonstrated that those with consistent daily pain experiences more accurately reported their pain on a retrospective survey than did those who experienced more heterogeneous pain symptoms (11). Similar observations have been made of objectively assessed phenomena, including cognitive and physical functioning, such that assessments collected in distraction-free clinical environments may poorly generalize to function in the context in which a person lives, and that variation in function may capture unique information in daily function and health. This is explored further in “EMAs of Cognition” below.

Recognizing the value of more frequent ecologically valid assessment of dynamic health determinants, research use of EMA methodologies has increased rapidly. Indeed, more than 1 700 publications have employed these methods to date (7). Notably, clinical use of EMA strategies remains limited, though many EMA protocols are similar to common self-monitoring strategies employed by cognitive-behavioral clinicians (12). We next provide a general overview of the reliability and validity of EMA techniques while highlighting advantages over traditional methodologies.

### EMA and Traditional “Gold Standard” Assessments

While EMA use is on the rise, many unanswered questions and opportunities specific to aging research remain. Smyth et al. recently published a review of pressing issues in EMA research (13), and many of the opportunities and challenges the authors outlined were echoed in presentation and conversation within the workshop. A recurring theme in the workshop was the desire to contrast EMA measures with contemporary infrequent measures of cognition, stress, affect, and other key causes and consequences of health in aging. As Stone et al. note, the very structure of many EMA protocols (eg, the collection of frequent assessments of phenomena over short timescales and in the real world) means the method captures information that is necessarily unique from information captured via summary measures, even when EMA measures are summarized at the individual level. This discrepancy was illustrated by Moore and colleagues (14), who conducted a randomized clinical trial of Mindfulness-Based Stress Reduction among emotionally distressed older adults (*N* = 67). Participants completed paper-and-pencil measures of mindfulness, depression, and anxiety pre- and postintervention. They also completed EMA surveys of the same items 3 times daily for 10 days before and following the intervention, with the exception being that EMA items queried the individuals’ current state, whereas paper-and-pencil measures asked about these constructs over the prior week. The researchers found that outcomes differed based on measurement method, such that significant changes in symptoms were only found when using the EMA data.

As underscored by Stone and colleagues (7) there is considerable room for focused research on the ways in which older adults interpret specific EMA items and draw upon information to answer a question, as well as the optimal methods for training participants to accurately and consistently respond to EMAs, and for reporting training protocols, as this is often lacking in EMA publications. From a clinical perspective, research is warranted investigating the utility of repeated versus infrequent assessment for providing better and more timely care. Put differently, it remains to be established whether the added complexity and burden of EMA meaningfully enhances quality of care. What is clear, however, is that the practice of contrasting EMA-collected data against traditional gold-standard summary self-reported measures is not likely a useful endeavor and instead researchers and clinicians would benefit by clarifying in what ways these methods capture unique and valuable information.

### Designing With Burden in Mind

An especially important consideration in the design of EMA research is participant burden. An EMA protocol must match the sampling frequency to the expected rate of fluctuation in the phenomenon of interest. For instance, capturing rapid changes in affect or stress states may require much more frequent sampling than measures of sleep quality. This must be held in balance with participant burden (15). Assessing too many constructs, utilization of lengthy questionnaires, or prompting participants too frequently risks high levels of attrition or missingness (13). This poses a second key challenge to the use of conventional “gold standard” measures of psychosocial determinants of health. Carefully elucidating what information is lost by gathering more frequent *brief* assessments of key phenomena remains a fruitful area for additional research, acknowledging that some constructs may be more amenable to brief report than others. A related and growing area of investigation concerns the extent to which sensor-equipped wearable devices may provide complimentary data to EMA (eg, estimates of sleep duration alongside EMA-reported perceived sleep quality) or may more accurately capture items historically captured by self-report (eg, physical activity volume). It is also notable that burdensome EMA schemes run the risk of becoming their own interventions, contributing to stress and reactivity, which may influence responses (16). Promisingly, well-designed EMA protocols consistently result in high completion rates across populations. Indeed, persons with schizophrenia and older adults with and without cognitive impairment average completion rates of ≥80% (17,18). In a newly published paper by the International Society of Clinical Trials Methodology Negative Symptoms Working Group, active and passive digital remote assessment technologies were evaluated for their readiness for implementation in pharmacological clinical trials targeting complex negative symptom domains. The Working Group concluded that EMA methods were ready for implementation as clinical endpoints in trials targeting negative symptoms, though the group emphasized the need to define EMA-specific gold standards and indeed to define processes for establishing these standards (19).

### EMAs of Cognition

A relatively recent area of EMA research concerns the collection of many repeated assessments of cognitive performance in the real world. Moreover, emerging research suggests that intraindividual variability in cognition may be a particularly sensitive marker of future cognitive decline (20–22) and fluctuations over time are hallmark features of some age-related neurodegenerative diseases (eg, sundowning, fluctuating attention in dementia with Lewy bodies). Schmitter-Edgecombe and colleagues (23) found that older adults’ intraindividual variability on a tablet-based n-back test (a measure of working memory) taken 4 times per day over 1 week was more strongly associated with self-reported functional status than an individual’s average performance on the task, or that same person’s lab-based global neurocognitive functioning. Aschenbrenner and colleagues (24) found that greater intraindividual variability on mobile cognitive tests taken 28 times over 1 week was associated with preclinical AD risk. A number of toolsets have emerged in recent years to facilitate the collection of cognitive performance measures repeatedly and in the field. For instance, NeuroUX (25), a platform to collect EMA, mobile cognitive testing, and passive sensor data, is now available in multiple languages and is being used at institutions across the United States as well as internationally. As another example, the Mobile Toolbox (26) arose from a collaboration between researchers at 8 institutions and offers a point-and-click system allowing for collection of mobile cognitive data on research participants.

Just as with the use of EMA methods to assess self-reported and subjective phenomena, the collection of cognitive performance within an EMA framework is a relatively new field of study, and there are several exciting and important opportunities for additional research in aging. First, there is considerable ongoing debate regarding the extent to which mobile collection of cognitive data should be expected to correlate with measures taken infrequently in a controlled environment. Studies directly comparing mobile versus controlled assessments of cognitive performance report correlations ranging from *r* = 0.48 to 0.53 (27,28). This is somewhat unsurprising: as noted previously, the repeated collection of data in the environments in which people live may capture unique information relative to infrequent assessment in controlled environments. A unique aspect of mobile cognitive assessment is the need to account for the effect of a high degree of practice on performance (29). However, the assessment of practice effects themselves may be valuable, as some researchers have found it to be a marker of cognitive decline (30). Second, as with the use of EMA to assess self-reported phenomena, developing and disseminating training paradigms that can efficiently prepare older adult participants of varying socioeconomic backgrounds and cognitive abilities to complete remote and repeated cognitive tests will be required for widespread clinical and research uptake of these procedures. Third, researchers should continue to work to disentangle the added information gleaned from repeated field assessments of cognition from the added noise of completing these tasks in potentially distracting contexts. Finally, working to develop easily understood and clinically valuable metrics derived from common cognitive assessments will be vital for establishing value for both clinicians and older adults around the use of EMA broadly, and EMA-measured cognition specifically.

### Confidentiality in the Clinical Use of EMA Techniques

An especially important consideration in deploying EMA methodologies in clinical care and research is data privacy and confidentiality. Indeed, this emerged as a consistent point of conversation across the workshop. Relative to in-clinic assessment, the remote collection and internet transmission of potentially sensitive psychophysiological data clearly increases the risk of loss of confidentiality. It is notable that at the present time, there are no standard technologies that are widely used for the collection of clinical EMA data. As such, risks to confidentiality may be heightened depending on factors such as which technologies are selected to collect psychophysiological EMA data, and technology literacy on the part of the researcher/clinician and patient. For instance, short messaging service (ie, SMS; text messaging) is widely used for prompting and patient data collection, though SMS data are not end-to-end encrypted and are stored in transmission with the wireless carrier. Similarly, integration of third-party technology providers (eg, wireless carriers, social media platforms, wearable devices) without transparent data-sharing policies increases the likelihood of patient data being shared or commercialized without their knowledge. To that end, it is important that researchers and clinicians interested in the use of EMA methodologies—or indeed the use of digital health technologies more broadly—collaborate with institutional privacy officers, information technology professionals, and legal professionals to select tools that meet institution privacy requirements and establish contracts with technology vendors to ensure data are handled securely.

## Considerations in Delivering Digital Technology-Supported Health Promotion Interventions to Older Adults

The same features that make smartphones, tablets, computers, and wearable devices attractive for data capture also make these digital health technologies valuable for the delivery of health-enhancing interventions. These technologies provide unprecedented access to individuals regardless of location or transport ability, the opportunity to dynamically tailor content to a person’s preferences and context, and the ability to offer much more frequent exposure to theory-based intervention tools in daily life. To this end, the second major theme of the workshop centered on challenges and opportunities in the design and implementation of technology-supported health behavior programming for older adults, and key design considerations that may help to avoid common pitfalls. First, speakers emphasized that while technologies may enhance access to healthcare, those technologies must be designed to meet the needs of the older adult, they must be perceived as useful and usable by the individual, and systems must be in place to distribute technologies and provide self-efficacy-oriented training and technical support designed to the diverse needs of older adults. Second, the downstream impacts of digital health technologies on related health determinants such as loneliness and isolation were highlighted, with speakers providing examples of socially supportive programming facilitated by technology. These 2 key topics are detailed further in the following sections.

### The Importance of Patient-Centered Design

Digital health technologies offer tremendous potential in terms of enhancing the health, well-being, and independence of aging adults, but this potential can only be realized if aging adults adopt and subsequently successfully engage with a given tool. Generally, data indicate that aging adults are receptive to using new technologies, are able to learn to interact with new technology systems and devices, and—most importantly—are more likely to adopt a broad array of new technologies if they perceive a technological tool as useful and contributing to quality of life (31). Further, although the uptake of technology has generally increased among older persons (typically defined as those 65 years of age and older), an age-related digital divide remains, especially among those with disabilities, of lower socioeconomic status (SES), or who live in rural locations. According to recent data from the Pew Internet Research Group (32), 75% of those aged 65 and older use the internet (as compared to 59% in 2014), and 64% indicate that they have home broadband. With respect to mobile devices, 61% of people ages 65 and older own a smartphone as compared to 83% of those ages 50–64 and 95% of those ages 30–49. Currently, only about 44% of adults aged 65+ report owning a tablet computer. In addition, digital literacy (ie, possessing the skills required to successfully interact with digital technology such as a mobile device) is lower among older adults, especially for minority populations and those with lower education and SES (33,34). Older adults also tend to have lower self-efficacy in their ability to interact with technology and less comfort using technology than do younger people (35). There is also an important rural–urban divide in technology usage wherein urban-dwelling older adults are more likely to use the internet, modern technological devices, and health technologies than are those living in rural communities (36–39).

Taken together, reduced access and greater prevalence of poor digital literacy and self-efficacy threaten to exaggerate health disparities among older adults. For example, data from the National Health and Aging Trend Study (40) demonstrated that although the use of telehealth services among older adults increased during the COVID-19 pandemic, older age and lower income were negatively associated with telehealth use. Importantly, however, in models adjusted for technology-enabling factors such as access to a digital device, online experiences, and learning a new technology skill during the pandemic, age and income were no longer significant predictors of telehealth usage. These data underscore the importance of providing older adults with *meaningful access* to digital technologies (ie, both access and the training required for skill and self-efficacy development). Common barriers to adoption of technology by aging adults include lack of access to and awareness of technology, lack of available training and technical support, cost, continual changes in technology, and usability issues. Importantly, meaningful access to digital health technologies requires understanding the functionality, limitations, and maintenance requirements of technology, having the requisite skills and training, and having access to technical support.

Discussion between workshop attendees highlighted the importance of centering older adults in the design and refinement process of the digital health tools they are expected to use. During tool development, it is important to incorporate as design partners older adults who are representative of the target user in terms of knowledge, skill, and self-efficacy for technology usage. This is especially important as the needs of the target user—including preferences, motor and perceptive needs, and understanding of user interface and experience norms—are likely to be quite different from members of the research or clinical development team. Moreover, factors such as one’s socioeconomic and cultural background may drive differences in preferences and needs within the older adult population. Next, as noted previously, purposeful training protocols designed to develop self-efficacy toward the specific tool should be developed with feedback from target users, and these protocols should be published to help to establish norms among geriatricians and researchers implementing digital health technologies. Notably, clinical or research staff that interact with users of the digital health tool must also be trained in how to communicate about the technology (eg, on the importance of positively framing the tool to avoid reinforcing potentially negative implicit associations with technology, on discussing privacy concerns). Finally, easily accessed technology support structures must be in place (2) to ensure that users receive several opportunities for low-stakes exposure to the technology and can have technical or user challenges addressed rapidly. Broadly, key considerations such as how will technologies be designed and adapted to the users, how will users be trained, and how will users be supported should be explicitly defined in funding applications, research protocols, and publications to establish norms in the research and clinical communities around the development of digital health technologies to support aging.

### Sustaining Social Support in Remotely Delivered Interventions

Another important consideration in the deployment of digital health tools is the extent to which they support or hamper other key inputs to well-being such as social connectivity. In the interest of cost-efficient scaling of health-enhancing programming, it is tempting to create fully automated (sometimes termed self-guided or unguided) eHealth and mHealth programs for older adults (41). There is a natural tension, however, between such an approach and the broader study of motivated behavior change, which places meaningful interpersonal connection at the hub of both well-being and behavior change and maintenance. As outlined by biopsychosocial models of healthy aging (6) and all widely used behavioral theories (eg, self-determination and social cognitive theories) (42,43), access to meaningful social connections is a core human need that underlies one’s ability to successful adopt and sustain health behaviors such as physical activity or healthy eating.

Physical activity behavior offers a useful example for understanding the interplay between social connection and motivated behavior change. Activity and social health are deeply connected constructs. Socially isolated older adults have higher levels of sedentary behavior (44), while enhancing an older adult’s social network is associated with improved functional and psychological health (45). Social support specific to physical activity (46), larger social networks, and engaging in social activities is associated with higher levels of physical activity in older adults (47). For example, a longitudinal study of close to 17 000 adults indicated that high social support during the COVID-19 pandemic was associated with 64% increased odds of sustaining physical activity (48).

In the context of activity promotion, mHealth technologies can provide objective real-time feedback on activity and sedentary behaviors, which is highly valuable as accurate self-monitoring is a requirement for effective behavioral self-regulation (49). With individuals tending to overestimate time spent in moderate-to-vigorous activity and underestimate time spent in sedentary behaviors (50,51), this feedback not only promotes self-awareness but also provides multiple opportunities to make necessary changes in behavior. Technologies can also cue goal successes in real time, which is vital for the development of self-efficacy (43,52), and can facilitate synchronous or asynchronous social connections at any time. Evidence suggests that access to social connections may underpin ongoing health technology usage among older adults (53). These connections provide myriad inputs to behavioral motivation, including (but not limited to): supportive interpersonal communication and feedback on activity progress; a sense of accountability; and an enhanced social environment and associated sense of security and relatedness (42,43,54,55). In a study of older adults living with Parkinson disease, there was a 31% increase in objectively measured physical activity after engagement in a socially supported physical activity intervention (56). Participants indicated that the addition of social engagement was more motivating than simply using an activity tracker alone to stimulate physical activity. A similar pattern was found in a study of older adults that compared the use of a mobile application (app) alone to the use of the app plus social interaction focused on physical activity (57). Those who had the combination of social interaction and the app significantly increased their mean steps/week, whereas those that only used the app did not. The COVID-19 pandemic provided a naturalistic environment to explore the relationship between physical activity, social connection, and digital health technology. A large cross-sectional study found that among adults over 55 years of age, those who usually exercised with friends and who did not engage with technology for exercise had a reduction in exercise levels during the pandemic (58).

Digital health technologies are purported to be a means to address health disparities and overcome common barriers to healthcare in underserved adult populations (59,60); however, social support may be even more integral to engage these groups. Adults from underrepresented groups identify social support and camaraderie as facilitators of enhanced physical activity (61,62). A study in adult Black women found that some participants needed added social support to maintain physical activity (63). Similarly, Black men reported that activity trackers with social components provided a means to extend their network of physically active individuals and sustain motivation for a healthy lifestyle (64). Within Hispanic populations health behavior may be defined by family needs and the influence of *familismo*, the cultural value of the family being of primary importance and influence (65). It is notable that many of these same principles may be of value across cultural groups. For instance, diabetes self-management interventions that have had a family-centered approach have been effective in enhancing physical activity and disease self-management (66,67). Digital health technologies should be culturally tailored to effectively engage these large segments of the population and help to bring our society closer to health equity.

Finally, it is notable that digital health technologies stand to affect—both positively and negatively—that patient–provider relationship, another key social connection affecting the health of older adults. Trust in the patient–provider relationship is associated with greater participation in, and satisfaction with, care (68–70). Easier access to telehealth care, especially for those with transport limitations or who are geographically isolated, may provide the opportunity for more frequent interaction and the development of more robust relationships with care providers. Likewise, access to objective information about a patient’s lived experience and health behaviors may contribute to more tailored and informed care. By contrast, access to more data may add burden to the patient–provider interaction (eg, discussing heart rate notifications given by a wearable device), and greater technology-related distraction or reduced face-to-face contact may hamper the development of trusting relationships. A review of 79 reviews describing the impact of digital health technologies on relationships and trust with providers (68) reported positive effects when these technologies supported personalized and collaborative care, and when they support and supplement rather than replace face-to-face interactions. Supporting recommendations we made above, the authors underscored the importance of user-centered design to ensure these technologies meet the preferences and needs of diverse patient populations and the implementation of training on effective technology use for both patients and providers.

## Future Directions and Research Opportunities

The emergence and rapid ubiquity of the internet and smartphone devices, and the downstream technologies facilitated by these platforms, represent very recent, socially and culturally transformative technological advancements. There is no doubt that these technologies will continue to change gerontology and geriatric medicine, and steps taken now can help to maximize benefit while avoiding worsening inequity and isolation. Speakers and attendees of the 2022 RCCN workshop on mHealth and digital health in aging highlighted several important future clinical and research directions:

1. *Refining objective and self-reported measures collected via EMA methodologies.* It is clear that the use of digital health technologies to assess self-reported and objective phenomena in daily life and in real time will become increasingly commonplace. To this end, it will be critical for clinical and research communities to identify reliable and valid EMA questions, prompting schemes, training methodologies, and measurement protocols. Moreover, given these complexities, it will be important to develop open and centralized platforms for researchers and clinicians to share their training and measurement protocols.

2. *Cultivating cultural and infrastructural support for successful uptake and maintenance of digital health technologies.* Ensuring uptake of digital health tools across experience levels and preferences requires that tools are perceived as useful and usable by the target user and are therefore designed in collaboration with representative target users. Those users must have access to training designed to impart the skills and efficacy required to adopt a new technology, and they must have access to technology support staff who are trained to address the needs of the target user in a way that sustains confidence and value toward the technologies. Indeed, all clinical or research staff interacting with individuals using digital health technologies should be trained on how to communicate about the technology in a proactive and positive manner to sustain value and self-efficacy. Integrating *digital navigators*, who are trained to provide technical support in healthcare, may be one fruitful avenue for minimizing clinical burden during deployment of new digital health technologies. This was underscored in a digital health action plan published by the World Health Organization (2), which emphasized that institutions must cultivate the cultural and infrastructural support needed to drive diverse uptake of new digital health tools (71).

As important is establishing utility, value, and support at the level of the health system. Presently, it is estimated that a primary care physician would require 26 hours per day to meet guidelines for providing preventive, acute, and chronic disease care and associated documentation and inbox management (72). Issuing digital health technologies, providing orientation and technology support, and providing necessary care and feedback therefore requires a team-based approach with funding, training, and clinical norms supporting this role on the clinical care team. Similarly, given the centrality of electronic health record (EHR) systems in clinical care, incorporation of digital health technologies into the EHR in ways that are low-burden and highly valued by the care team will also be a requirement for widespread clinical integration (73).

3. *Sustaining interdisciplinary collaboration.* It is important to recognize that the development, deployment, and maintenance of useful digital health technologies is a multidisciplinary endeavor. Clearly collaborative computer scientists, human factors engineers, and designers who are well-versed in clinical and behavioral needs will continue to play a critical role in the development of new and more effective digital health technologies. Clinicians, implementation scientists, and health systems professionals are required to understand how to integrate EMA and health-promoting technologies into current and emerging care models. Statistical, data science, and bioinformatic experts are required to develop and share techniques for deriving meaningful results from multiple high-resolution data streams capturing unique self-reported and objective phenomena (74). This expertise is especially important for navigating the benefits and pitfalls associated with increasing integration of artificial intelligence and machine learning models in digital health. Additionally, given that technologies should be developed and refined to meet the needs of the local population, it should be expected that tools and measurement protocols may differ by location. Therefore, methods for harmonizing data from unique measures collected using unique protocols and in unique environments are required. Finally, experts in the behavioral sciences play an important role in considering the myriad indirect effects of supplying novel technologies on overall health and well-being (eg, by providing access to new social connections vs incentivizing isolation via automated delivery of care).

## Conclusion

Digital health technologies will play an increasingly important role in gerontology and geriatric medicine, especially for understanding the dynamic changes in health status with aging. In many ways, the application of these tools is in its early days, and workshop attendees identified several key challenges and research opportunities, including (i) the need to identify valid and reliable measures that leverage EMA methodologies, recognizing such an approach captures unique information relative to the traditional use of infrequent summary questionnaires; (ii) the need to develop infrastructure to train and support both older adult participants/patients and staff in the use of these measurement and intervention technologies; (iii) the importance of assembling interdisciplinary teams dedicated to advancing the use of digital health tools in research and clinical practice; and (iv) the importance of following a user-centered design philosophy in the creation, evaluation, and implementation of digital health technologies.

## Data Availability

This article does not report data and therefore the preregistration and data availability requirements are not applicable.
